# Ultrasound Evaluation of Cosmetic Changes in Nails: A Case Series and Literature Review

**DOI:** 10.7759/cureus.87752

**Published:** 2025-07-11

**Authors:** Claudia Gonzalez, Natalia Bocanegra-Oyola, Valeria Duque-Clavijo, Adriana Patricia Cruz Garnica, Mario Mahecha-Carvajal

**Affiliations:** 1 Radiology, Highly Specialized Ultrasound Center, Bogota, COL; 2 Epidemiology, Universidad del Rosario, Bogota, COL; 3 College of Medicine, Universidad de los Andes, Bogota, COL; 4 Dermatology, Hospital Universitario La Samaritana, Bogota, COL; 5 Radiology, Universidad de los Andes, Bogota, COL

**Keywords:** acrylic nails, gel nail polishes, methacrylate deposits, nail cosmetic-related complications, nail ultrasonography, porcelain nails

## Abstract

Background: The use of semi-permanent nail polishes and acrylic nails is rising due to cosmetic appeal, but may lead to underrecognized complications. High-frequency ultrasound (HFUS) offers a non-invasive method for assessing nail structural changes beyond clinical examination.

Objectives: To describe ultrasound findings in patients with complications from semi-permanent polishes and acrylic nails and correlate them with clinical presentations.

Methods: This descriptive study included 13 women over six months who used semi-permanent polishes or acrylic nails and presented with nail alterations. HFUS was performed using 8-22 MHz probes to assess nail structures in grayscale and Doppler. Defined criteria identified brittle nails, onychomadesis, ventral pterygium, and onychocryptosis. Data were analyzed descriptively.

Results: Among 13 patients, 69.2% used semi-permanent polishes and 30.8% used acrylic nails. Brittle nails were most common overall (30.8%), primarily in semi-permanent polish users (44.4%), while ventral pterygium predominated among acrylic nail users (50%). HFUS revealed methacrylate deposits within the intermediate plate in 55.6% of semi-permanent polish users. Increased vascularization on Doppler was seen in all cases, suggesting chronic inflammatory processes. Multiple structural alterations were frequently observed in individual patients.

Conclusion: HFUS effectively identified structural and vascular complications linked to cosmetic nail procedures, often detecting subclinical changes. Methacrylate incorporation into the nail plate may underlie many findings in semi-permanent polish users, while matrix atrophy and ventral pterygium were more typical of acrylic nails. These results highlight the importance of patient education and dermatologic monitoring. Larger studies are warranted to confirm these associations.

## Introduction

Nail structure and integrity can be influenced by multiple factors, including physiological processes and exposure to chemical agents. Natural factors influencing nail health include aging and microbial infections, whereas chemical factors encompass exposure to solvents found in nail polish and household detergents [1]. Nail damage resulting from manicuring may arise from both the tools and the products employed; commonly implicated products include nail polishes, sculptured nails, preformed nails, and, more recently, long-lasting gel nail polishes (GNP) [2,3]. These techniques differ in both composition and method of application, as outlined below. Traditional nail polishes consist of lacquer-based formulas that dry through solvent evaporation, offering temporary color without structural reinforcement. Sculptured nails involve molding acrylic material directly onto the nail plate or a form to create custom extensions, while preformed nails are plastic tips adhered with cyanoacrylate glue for immediate length and shape. GNPs, which combine gel and polish elements, are cured under ultraviolet (UV) or light-emitting diode (LED) light, resulting in a durable, glossy finish that can last for several weeks. This article focuses on the complications associated with GNPs and acrylic nails [1-3].

Nail cosmetic-related complications vary according to the type of product used. The most commonly identified adverse effects associated with specific nail cosmetic modalities, for acrylic and porcelain nails, were predominantly paresthesias, allergic contact dermatitis (ACD), psoriasiform onychodystrophy, and traumatic onycholysis. Light-curing gel systems were linked to allergic contact dermatitis and mechanical injury to the nail plate. Adhesive nail systems, including press-on, glue-on, and silk wraps, were more frequently associated with allergic contact dermatitis, nail plate thinning, onycholysis, and pitting. Gel polishes, encompassing gel manicures, long-lasting, semi-permanent, and UV-cured formulations, were most commonly related to allergic contact dermatitis, mechanical damage, and ultraviolet-induced lesions [3].

## Materials and methods

Objectives

General Objective

To describe the ultrasound findings in patients with complications associated with using acrylic nails and semi-permanent nail polishes.

Specific Objectives

1. Identify the most frequent ultrasound characteristics of complications related to the use of acrylic nails and semi-permanent nail polishes.

2. Determine the types of clinical complications observed in patients using acrylic nails and semi-permanent nail polishes, correlating them with the ultrasound findings.

Study design

A descriptive observational study was conducted to document nail complications associated with the use of semi-permanent nail polishes and acrylic nails in a specific population. The study did not aim to establish causal relationships but rather to describe the ultrasound characteristics of the observed complications.

Study population

The inclusion criteria for the study involve women over 18 years old who have regularly used semi-permanent nail polishes or acrylic nails within the past six months, and who are seeking dermatological consultation for any nail alterations. Exclusion criteria include individuals with pre-existing nail conditions unrelated to the use of nail polishes, as well as those diagnosed with allergies to nail products. 

Ultrasound evaluation and definitions

All patients were evaluated using high-frequency ultrasound (HFUS) with a Hockey Stick 8-18 Multi-Frequency MHz (GE Venue system, GE HealthCare, Chicago, IL, USA) and Hockey Stick 18-22 Multi-Frequency MHz (Aplio 200 system, Canon Medical Systems, Otawara, Japan), following the guidelines established for such studies in previous publications [4-6]. All affected nail units were evaluated using grayscale and color Doppler imaging, and imaging records were obtained in both axial and longitudinal planes for each unit in all cases.

Specific ultrasound criteria were used to identify complications requiring detailed sonographic characterization: Brittle nails were described as thinning of the nail plate with partial loss of the dorsal plate, primarily due to thinning of the intermediate layer, with preservation of the nail bed and matrix. Onychomadesis was defined as the separation of the nail plate from its origin at the matrix, best visualized on longitudinal scans, allowing precise measurement in millimeters. Ventral pterygium was identified as a hyperechoic structure adherent to the ventral plate, extending to the epithelial surface of the hyponychium. Onychocryptosis was recognized by a trilaminar hyperechoic image corresponding to a fragment of the nail plate embedded within the medial or lateral nail folds, surrounded by a peripheral hypoechoic halo indicating a foreign body reaction.

Sample size

A total of 13 patients were included over a period of six months, selected for convenience from a dermatology clinic specialized in nail alterations. 

Given the exploratory design of this study, priority was given to obtaining detailed and contextual data from a limited number of participants. Logistical factors, such as the availability of patients meeting strict inclusion criteria, significantly influenced the sample size. Additionally, restricted access to the target population, along with time and resource constraints, further limited the number of participants enrolled. Despite the small sample size, the findings provide valuable preliminary evidence that may inform and justify future studies with larger cohorts.

Data collection

Data collection was carried out using a database of ultrasound findings. Only patients who met the inclusion criteria were included, while those who did not meet these criteria were excluded from the study.

Data analysis

The obtained data were processed using descriptive analysis. Absolute frequencies were calculated for the observed complications, and categorical variables, such as the type of polish and the observed complications, were analyzed using percentages. The results were presented in tables summarizing the descriptive findings and their frequency in relation to the type of polish. Given the sample size, more complex statistical analyses to determine associations or causality were not feasible.

Ethical consideration

The study was conducted in accordance with the ethical standards of the Declaration of Helsinki. Informed verbal consent was obtained from all patients prior to ultrasound examination. Patient data were anonymized to ensure confidentiality, with secure storage and access limited to authorized personnel.

## Results

**Figure 1:**
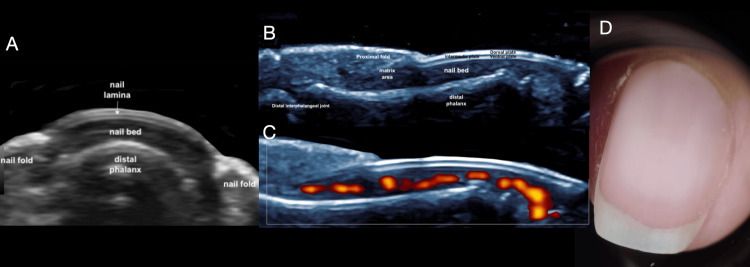
Normal nail anatomy on ultrasound and dermoscopy. (A) Grayscale axial image showing the normal ultrasonographic anatomy of the nail. (B) Longitudinal grayscale image depicting the normal anatomy of the nail. (C) Longitudinal power Doppler image demonstrating normal vascularization of the nail. (D) Dermoscopy image showing the appearance of the normal nail lamina.

**Figure 2:**
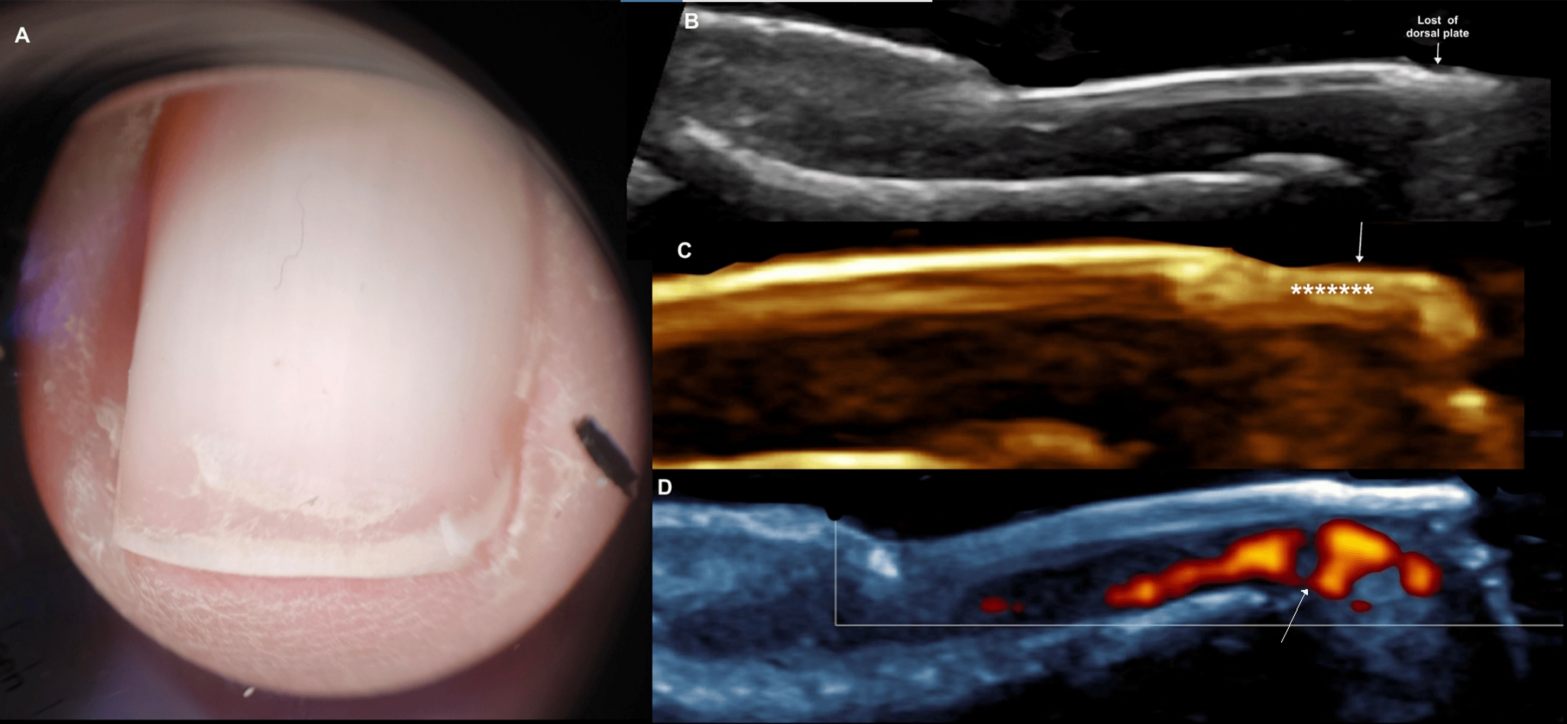
Fragile nail syndrome following gel polish removal: clinical and ultrasound findings. A 46-year-old female patient, a frequent user of gel nail polish, which has been removed, is now developing Fragile nail syndrome following gel polish removal. (A) Clinical dermatoscopic image showing onychoschizia and lateral folds desquamation (paronychia). (B) Longitudinal grayscale ultrasound image demonstrating loss of the distal dorsal plate corresponding to the area of onychoschisis. (C) Magnified longitudinal grayscale image of the nail lamina: the white arrow indicates loss of the dorsal plate, while asterisks highlight hyperechogenicity and loss of definition in the intermediate and ventral plates. (D) Longitudinal power Doppler image with white arrow indicating a focal area of increased vascularization immediately beneath the site of dorsal plate loss, reflecting a significant inflammatory component associated with gel polish removal.

**Figure 3:**
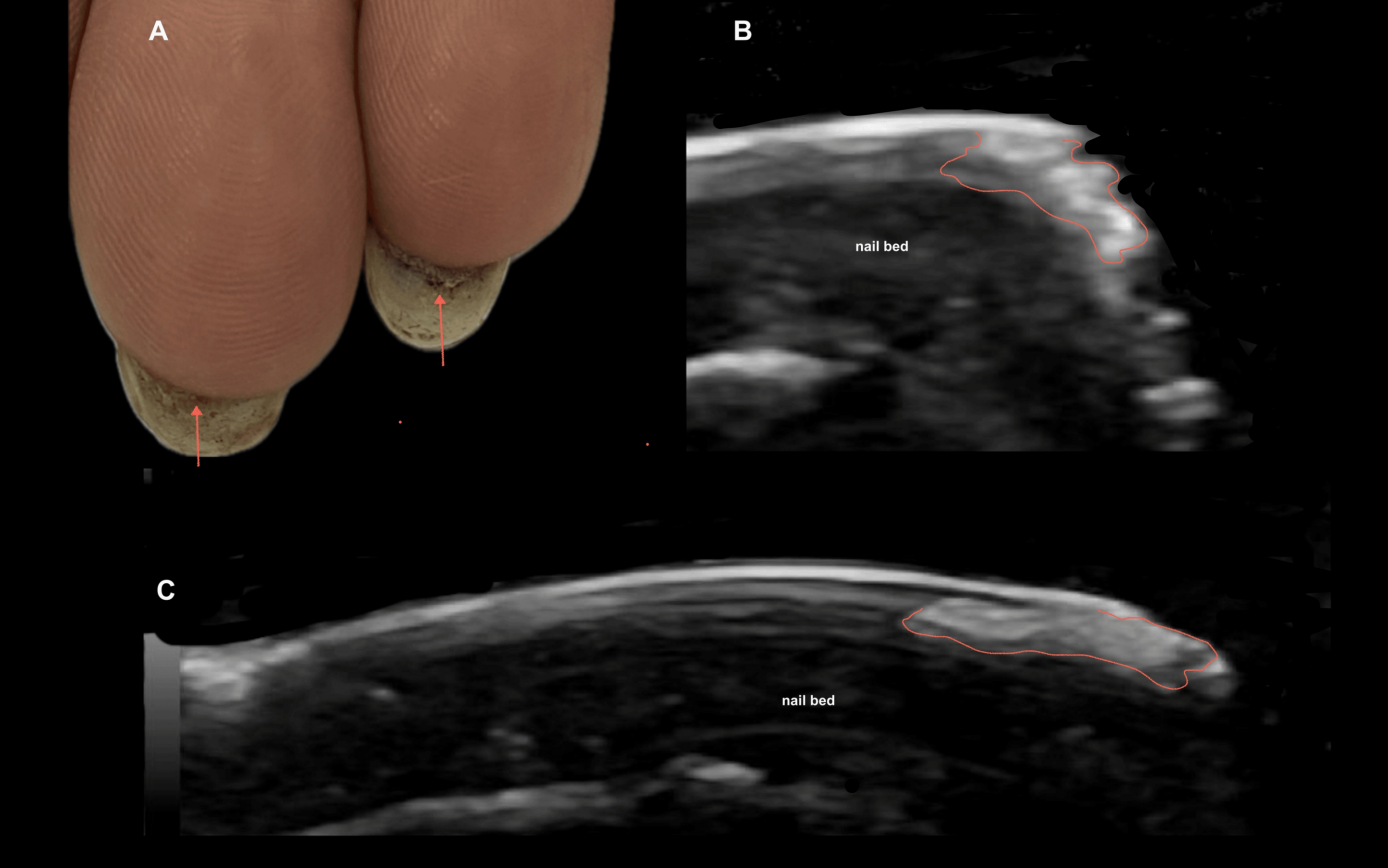
Pterygium inversum unguis in a gel polish user: clinical and ultrasonographic correlation. Female patient of 19 years of age permanent user of gel nail polish. (A) Clinical image showing pterygium inversum unguis (PIU) indicated by red arrows on the third and fourth fingers. (B) Magnified longitudinal grayscale ultrasound image of the distal third of the third ungual unit, delimited in red, showing defined, irregular hyperechoic tissue corresponding to PIU, where the hyponychium adheres to the ventral nail plate. (C) Longitudinal ultrasound image of the fourth ungual unit, also delimited in red, demonstrating hyperechoic tissue consistent with PIU, which appears more longitudinal in this finger. Ultrasound allows a precise definition of the extent of PIU.

The study included 13 female patients; 30.8% (n=4) used acrylic nails and 69.2% (n=9) used semi-permanent polishes. For a better understanding of the ultrasound images of the abnormal findings, the ultrasound appearance of the normal nail is shown in Figure 1 [1]. The most common clinical diagnosis among semi-permanent nail polish users was brittle nails, observed in 44.4% (n=4) of this subgroup, corresponding to 30.8% (n=4) of the entire study population, thus representing the most frequent diagnosis overall (Figure 2) [2]. In contrast, among patients with acrylic nails, the predominant diagnosis was ventral pterygium, present in 50% (n=2) of this subgroup (Figure 3) [3]. The distribution of other clinical diagnoses observed, including pseudopsoriasis, trachyonychia, and onychoschizia, is presented in Table 1.

**Table 1:** Clinical diagnoses stratified by type of cosmetic nail procedure. Data are presented as the number of patients (percentage). Percentages are calculated within each column. No statistical test was performed.

Clinical diagnosis	Cosmetic procedure		Total (n=13)
	Acrylic nails 30.8% (n=4)	Semi-permanent polish 69.2% (n=9)	
Brittle nails	0 (0.0%)	4 (44.4%)	4 (30.8%)
Onychomadesis	0 (0.0%)	3 (33.3%)	3 (23.1%)
Pseudopsoriasis	1 (25.0%)	1 (11.1%)	2 (15.4%)
Trachyonychia and onychoschizia	1 (25.0%)	0 (0.0%)	1 (7.7%)
Ventral pterygium	2 (50.0%)	1 (11.1%)	3 (23.1%)

Ultrasound findings showed that the same patient could present multiple alterations (Table 2).

**Table 2:** Main characteristics of the study population.

Cosmetic procedure	Clinical diagnosis	Sex	Age	Grayscale ultrasound	Doppler	Other ultrasound findings
Semi-permanent polish (already retired)	Brittle nails	Female	18	Thinning of the distal third of the nail plate. Loss of the dorsal nail plate.	Increased vascularization	Onychocryptosis
Semi-permanent polish (already retired)	Brittle nails	Female	46	Thinning of the distal third of the nail plate. Loss of the dorsal nail plate. Matrix and nail bed atrophy. Perionyxis.	Increased vascularization	No
Semi-permanent polish (Already retired)	Brittle nails	Female	44	Thinning of the distal third of the lamina. Loss of the dorsal nail plate.	Increased vascularization	No
Acrylic nails	Ventral pterygium	Female	23	Acrylic nail, thinning of the nail bed, matrix atrophy. Ventral pterygium.	Increased vascularization	No
Acrylic nails	Ventral pterygium. Perionyxis of the nail fields	Female	25	Acrylic nail. Thinning of the nail bed. Matrix atrophy. Ventral pterygium. Perionyxis.	Increased vascularization	No
Acrylic nails	Pseudo psoriasis	Female	59	Increased thickness of the nail bed. Acrylic nails. Increased matrix.	Increased vascularization	No
Semi-permanent polish	Onychomadesis	Female	33	Increased thickness of the intermediate nail plate. Hyperechoic foci in the intermediate nail plate due to methacrylate deposits. Onychomadesis.	Increased vascularization	No
Semi-permanent polish	Onychomadesis	Female	31	Increased thickness of the intermediate nail plate. Hyperechoic foci in the intermediate nail plate due to methacrylate deposits. Onychomadesis.	Increased vascularization	No
Semi-permanent polish	Onychomadesis	Female	21	Increased thickness of the nail bed. Hyperechoic foci in the intermediate nail plate due to methacrylate deposits.	Increased vascularization	No
Semi-permanent polish	Ventral pterygium	Female	19	Increased thickness of the intermediate nail plate. Hyperechoic foci in the intermediate nail plate due to methacrylate deposits. Ventral pterygium.	Increased vascularization	No
Semi-permanent polish (already retired)	Pseudo psoriasis	Female	59	Increased thickness of the nail bed. Hyperechoic foci in the intermediate nail plate due to methacrylate deposits. Beau's lines.	Increased vascularization	No
Semi-permanent polish (already retired)	Brittle nails	Female	55	Thinning of the distal third of the nail plate. Loss of the dorsal nail plate.	Increased vascularization	No
Acrylic nails (already retired)	Trachyonychia and onychoschizia	Female	22	Increased thickness of the intermediate plate. Increased thickness of the nail bed. Onychomadesis.	Increased vascularization	No

**Figure 4:**
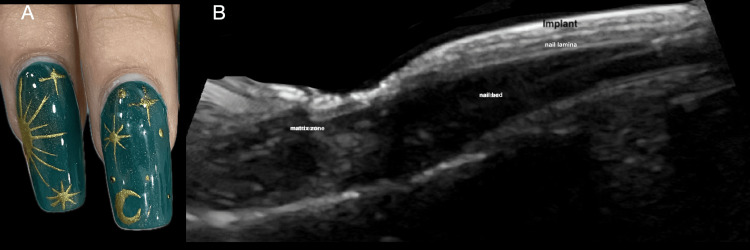
Ultrasound appearance of acrylic nails. (A) Clinical image of a patient with a manicure featuring acrylic nails. (B) Longitudinal high-resolution ultrasound image showing the acrylic nail implant as a moderately hyperechoic structure relative to the native nail lamina, located immediately above it.

**Figure 5:**
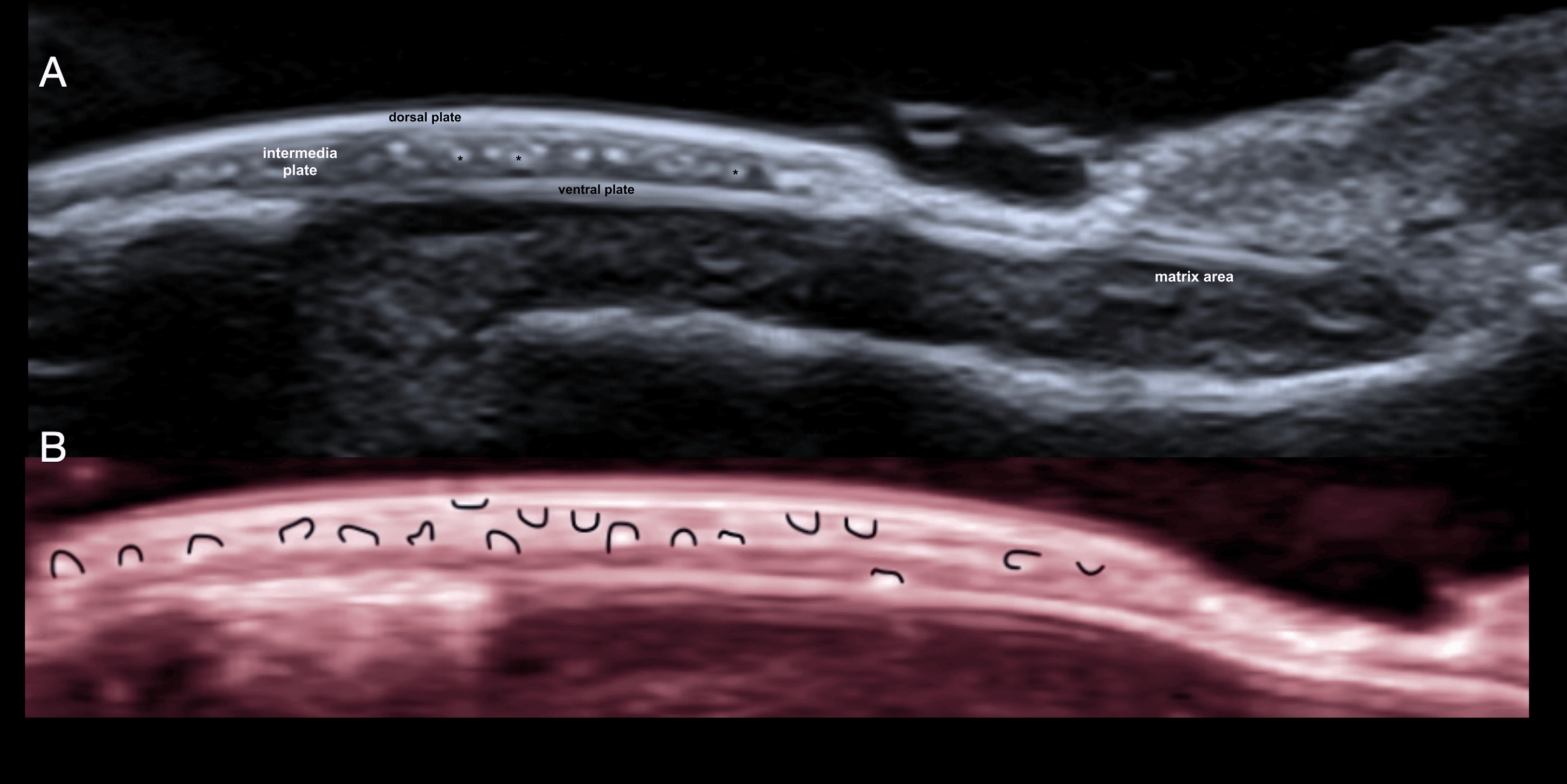
Methacrylate deposits in gel nail polish users: grayscale and color mapping. (A) Longitudinal grayscale image of nails with a gel nail polish manicure, showing an increase in the thickness of the intermediate plate. Asterisks indicate some of the multiple hyperechoic foci corresponding to methacrylate deposits. (B) Color-coded map (red) highlighting the random distribution of methacrylate deposits, visualized as multiple hyperechoic foci outlined in black.

**Figure 6:**
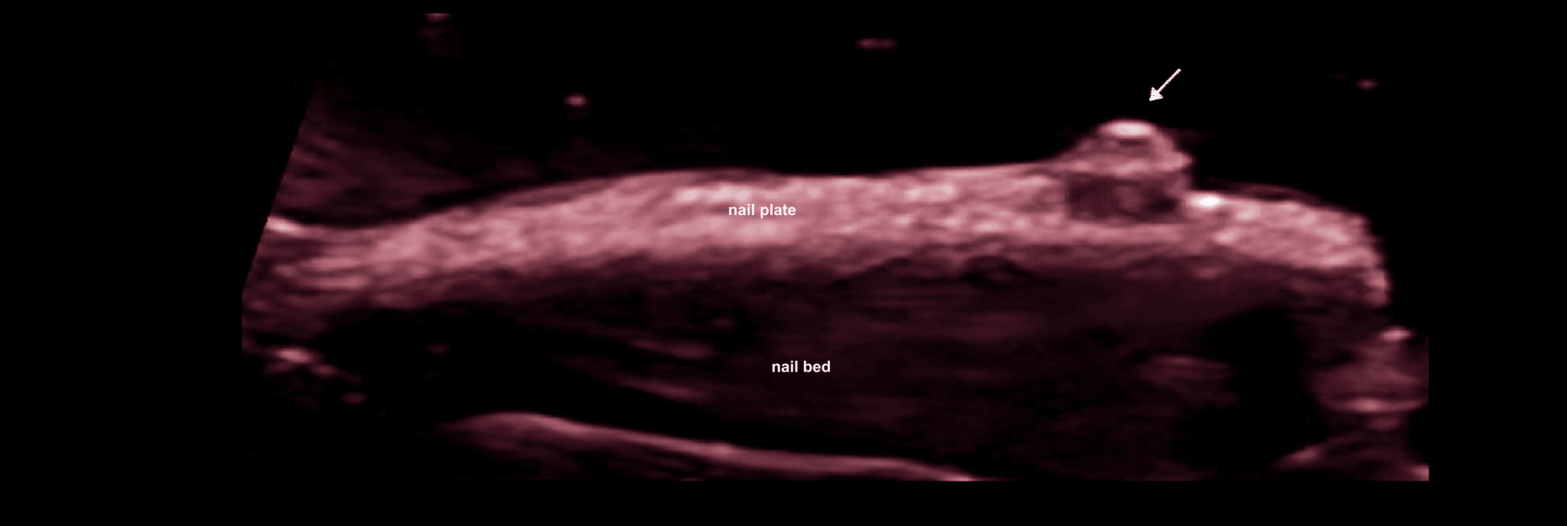
Decorative plastic elements and methacrylate residue in gel manicures. Longitudinal grayscale image. The white arrow indicates a decorative plastic element adhered to the nail plate. There is a loss of the trilaminar pattern of the nail plate and the presence of extensive, randomly distributed hyperechoic deposits corresponding to methacrylate from the gel nail polish.

**Figure 7:**
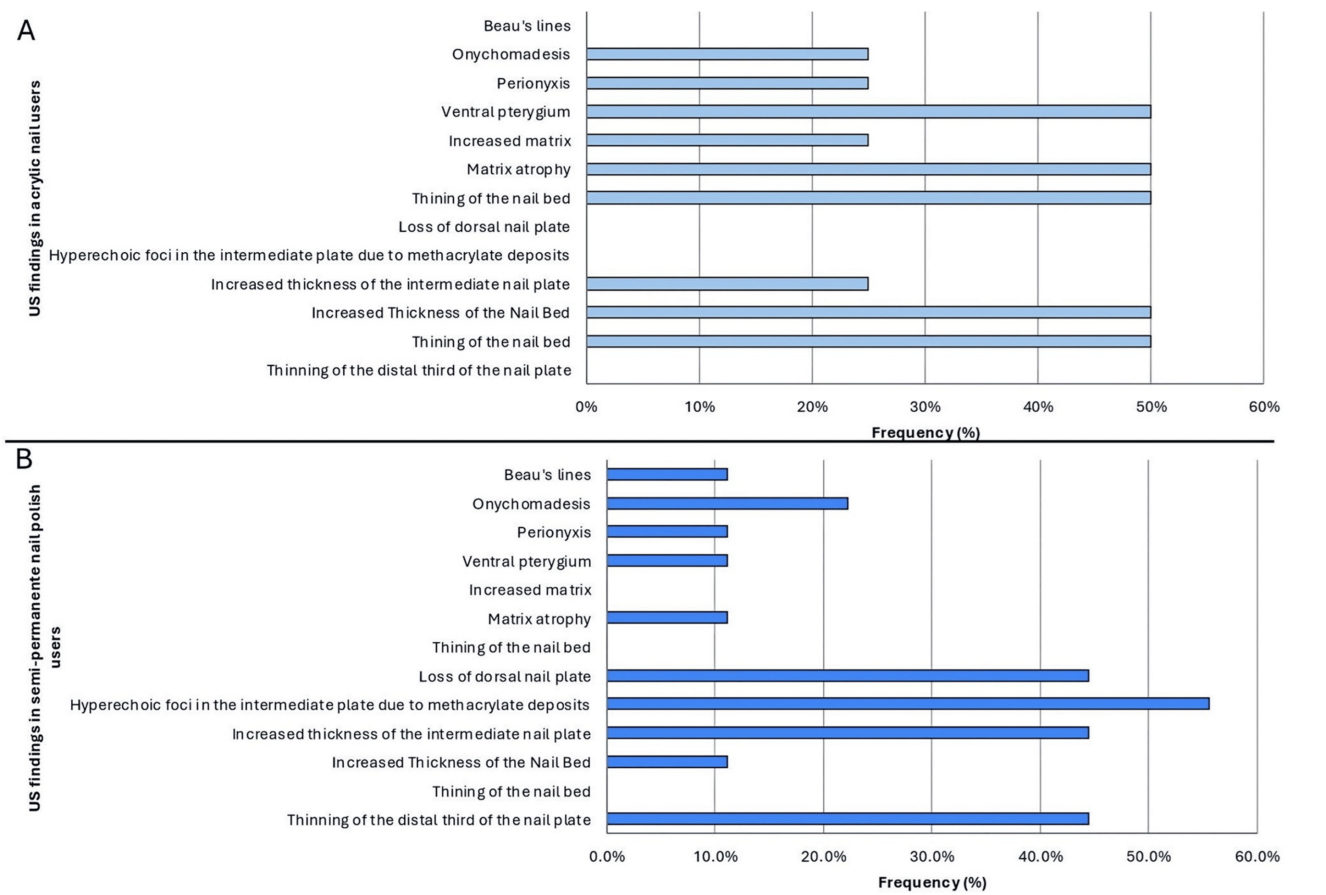
Ultrasound findings stratified by type of cosmetic nail procedure. (A) Frequency of grayscale ultrasound findings in individuals with acrylic nails (n = 4). (B) Frequency of grayscale ultrasound findings in individuals with semi-permanent polish (n = 9). Values represent the percentage of patients exhibiting each ultrasound feature. No statistical comparisons were performed due to the small sample size.

The ultrasound appearance of acrylic nails is easily recognizable (Figure 4). In users of semi-permanent nail polish, multiple ultrasound abnormalities were often observed simultaneously, meaning that individual patients frequently exhibited more than one finding. The most common alteration was the presence of methacrylate deposits in the intermediate plate, identified in 55.6% (n=5) of cases (Figure 5). Decorative elements made of methacrylate, glued onto the nail surface, were also easily visualized by ultrasound (Figure 6). Additional findings included thinning of the distal third of the nail plate (44.4%, n=4), loss of the dorsal plate (44.4%, n=4), and increased thickness of the intermediate plate (44.4%, n=4). Less frequent findings were onychocryptosis, paronychia, matrix atrophy, and ventral pterygium, each observed in 11.1% (n=1) of cases; onychomadesis in 22.2% (n=2); and Beau’s lines in 7.7% (n=1) (Figure 7A).

Similarly, individuals using acrylic nails often exhibited multiple ultrasound findings simultaneously, meaning that a single patient could present with more than one alteration. The most frequent findings were thinning of the nail bed, increased thickness of the nail bed, matrix atrophy, and ventral pterygium, each observed in 50% (n=2) of these patients. Additionally, increased thickness of the intermediate nail plate, increased matrix thickness, paronychia, and onychomadesis were each observed in 25% (n=1) of patients (Figure 7B).

**Figure 8:**
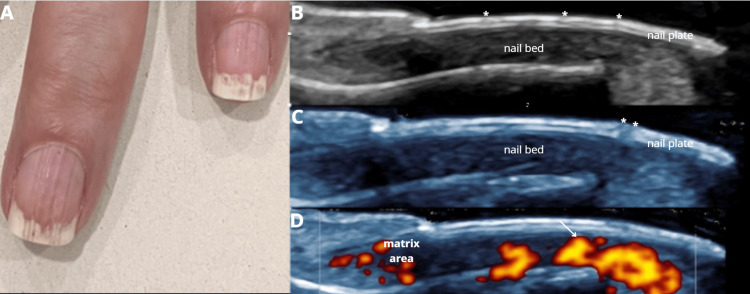
Chronic use of acrylic nails mimicking psoriasis: pseudopsoriatic ultrasound pattern. (A) Clinical image of a 59-year-old female patient with uninterrupted use of acrylic nails for three years. Following removal, clinical findings, including roller-coaster transverse onycholysis with a proximal erythematous border and onychorrhexis, raised suspicion of psoriasis, which required exclusion. (B) and (C) Longitudinal grayscale ultrasound images showing preserved trilaminar architecture of the nail plates. However, hypoechoic vertical and oblique bands (indicated by asterisks) traverse the entire lamina, consistent with chronic microtrauma. No ultrasound signs of psoriasis are present, such as hyperechoic foci with posterior acoustic shadowing or thickening of the nail bed. (D) Longitudinal power Doppler image showing hypervascularization (white arrow) beneath the ventral plate, reflecting chronic inflammation. These findings are consistent with pseudopsoriatic changes secondary to chronic acrylic nail use.

**Figure 9:**
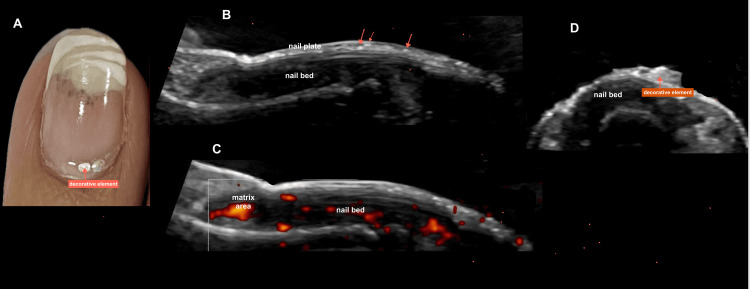
Pseudopsoriasis in chronic gel polish use: clinical and imaging correlation. Female patient, 59 years old, with continuous use of gel nail polish for two years. (A) Clinical image showing a gel manicure with decorative elements adhered to the nail plate (red arrow), along with onycholysis and splinter hemorrhages. (B) Longitudinal grayscale ultrasound image demonstrating loss of the trilaminar pattern of the nail lamina, with irregular, horizontally oriented hyperechoic lines. Red arrows indicate punctiform hyperechoic foci corresponding to methacrylate deposits within the lamina. (C) Longitudinal power Doppler image showing a mild increase in vascularization in the matrix area, indicating inflammatory activity. (D) Axial grayscale ultrasound image with a red arrow pointing to the decorative element adhered to the nail plate. The clinical and imaging findings are consistent with pseudopsoriasis.

A diagnosis that deserves special consideration for the implications of making an accurate diagnosis is pseudosporiasis. This was observed in two patients of our total population, being more frequent in users of acrylic nails than in users of semi-permanent enamels (Figures 8, 9). The precise distribution is shown in Table 1.

**Figure 10:**
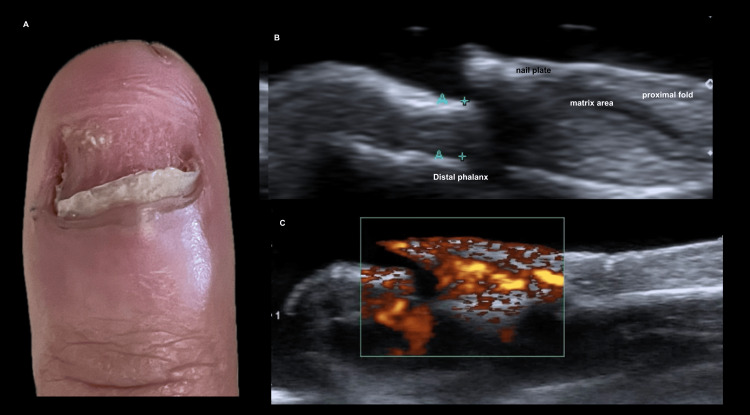
Nail plate destruction and chronic inflammation after acrylic nail removal. (A) Clinical image of a 22-year-old female patient showing near-complete loss of the nail plate following traumatic removal of acrylic nails. (B) Longitudinal grayscale ultrasound image demonstrating a residual hyperechoic segment of the nail plate with loss of normal ungual morphology. There is increased thickness of the proximal nail bed and matrix area (measured between blue calipers, A+). The hypoechoic nail bed also appears thickened, consistent with chronic inflammation. (C) Longitudinal power Doppler image showing intense vascularization, reflecting the degree of chronic inflammation.

Doppler study revealed increased vascularization in 100% of patients (n=13), regardless of cosmetic application type (Figure 10).

## Discussion

Currently, high-resolution ultrasound is considered the modality of choice for studying ungual pathology [7,8]. This is because its high spatial resolution allows an almost histological definition of the components of the ungual apparatus. The use of Doppler analysis enables real-time identification of nail vascularization without the need for intravenous contrast, allowing the assessment of the degree of vascularization of different lesions, their inflammatory activity, and therapy follow-up in inflammatory diseases such as lichen planus and psoriasis [9,10].

As mentioned, the excellent spatial resolution of HFUS allows for the visualization of elements used in cosmetic nail procedures [11], as well as the identification of pathological structural changes and complications arising from these elements. The normal ultrasonic appearance of acrylics is that of a linear band that is typically more echogenic than the native nail plate and is positioned immediately above the dorsal plate.

Gel nail polishes (GNPs) have gained significant attention in recent years due to their cost-effectiveness, prolonged durability, and ease of application [3]. These formulations typically consist of 75-95% urethane methacrylates, 0.75-1.25% polymerization catalysts (such as dimethyltolylamine), 1-4% photoinitiators, and various coloring agents. The appearance of these gel nail polishes in high-resolution ultrasound is primarily determined by the dominant presence of methacrylates, which are visualized as punctiform and hyperechoic foci. Interestingly, these foci are integrated into the dorsal, intermediate, and ventral plates of the nail and are randomly distributed along the nail plate. Notably, the ultrasound appearance of these methacrylates on the nails has not been previously described in the literature. However, their appearance has been documented in other areas of the body [12,13].

In addition to the use of gel polish for manicure procedures, various decorative elements, such as plastic or methacrylate, can be adhered to the surface of the dorsal plate. These elements are readily identifiable with high-resolution ultrasound.

Newly developed hybrid gel polishes and nail lacquers additionally contain cellulose derivatives, plasticizers, and organic solvents [2]. Their polymerization and hardening are achieved through photoactivation, which occurs via exposure to ultraviolet (UV) light for approximately two minutes or to light-emitting diode (LED) sources for 30 seconds. Although UV lamps are more commonly used due to their lower cost [2,14], GNPs have been associated with various adverse effects. These effects are primarily categorized into mechanical and traumatic nail disorders, allergic contact dermatitis (ACD), and ultraviolet radiation (UVR)-induced lesions [3].

Mechanical and trauma-induced nail damage primarily manifests as pterygium inversum unguis (PIU), followed by pseudoleukonychia, lamellar onychoschisis, brittle nails, thinning of the nail plate, xanthonychia, claw nails, pitting, and trachyonychia [3]. PIU is characterized by the abnormal adherence of the hyponychium to the ventral surface of the nail plate, resulting in an unsightly and often painful condition. In our population, PIU was the second most common clinical complication, affecting 50% (n=2) of acrylic nail users and 11.1% (n=1) of semi-permanent nail polish users. This clinical finding was confirmed in all cases by HFUS.

Nail plate thinning has been assessed via ultrasound, and in cases of PIU, reflectance confocal microscopy has revealed nucleated cells and keratohyalin granules [9,10]. This condition is hypothesized to be potentially associated with the use of LED photoinitiators [10]. Lesions resolved completely within one to three weeks, whereas claw nail deformities required more than four months for full recovery [3].

Brittle or fragile nails are a relatively common condition that can be idiopathic or secondary to various factors, such as trauma induced by cosmetic procedures [15]. In our patient series, brittle nails were observed in four patients, all of whom used gel nail polish. As previously noted, the methacrylate in gel nail polish integrates into all layers of the ungual lamina, primarily the intermediate plate. Consequently, removal of the polish requires aggressive mechanical and chemical methods to reach the intermediate plate, often resulting in onychorrhexis, lamellar onychoschizia, and other changes characteristic of fragile nails.

HFUS provides clear visualization of the extent of dorsal lamina loss in cases of onychoschizia, allows quantification of intermediate lamina thinning (a consistent ultrasound finding in these patients), and assesses nail unit vascularization, reflecting both chronic and acute inflammatory components of the condition.

These mechanical alterations have been predominantly associated with the application and removal procedures of cosmetic nail polishes, which involve the use of various chemical agents [16-18]. These agents have also been implicated in the development of paronychia in some cases. Additionally, mechanical manipulations may contribute to the onset of onycholysis, subungual hyperkeratosis, and, in certain instances, PIU, due to damage sustained by the hyponychium [2,3]. The removal process can be so aggressive that it may cause near-complete destruction of the nail plate.

A clinical pattern specifically observed in patients with acrylic nails is psoriasiform onychodystrophy [3], characterized by subungual hyperkeratosis, onycholysis, and periungual dermatitis secondary to the application of artificial nails and gel nail polish (Figure 8). However, periungual dermatitis is not universally present [19]. Therefore, accurate differential diagnosis is essential, as other characteristic features of nail psoriasis are typically absent [20]. This condition may be associated with koebnerization [21].

In our series, pseudopsoriasis was observed in 25% (n=1) of acrylic nail users and 11.1% (n=1) of gel nail polish users. High-frequency ultrasound (HFUS) is particularly useful in differentiating pseudopsoriasis from true nail psoriasis. The ultrasonographic characteristics of nail psoriasis have been extensively described and histopathologically validated in multiple studies [22,23]. Five ultrasonographic stages are recognized based on severity, and HFUS can also detect early or subclinical psoriatic arthropathy [24].

In pseudopsoriasis, HFUS reveals an absence of classic psoriasis findings; instead, morphological alterations of the nail lamina associated with microtrauma predominate, along with residual methacrylate deposits within the lamina.

Acrylic monomers are the primary agents responsible for most cases of acrylate-induced allergic contact dermatitis (ACD). Among these, 2-hydroxypropyl methacrylate (HPMA) and 2-hydroxyethyl methacrylate (HEMA) are the most commonly implicated acrylates, with their allergenic potential well documented in affected individuals. HEMA is particularly notable as the most frequent sensitizing methacrylate, identified in 85.3% of acrylate sensitization cases [25]. This compound is consistently present in lacquers used for gel nails, whereas its presence in acrylic nail formulations varies [19].

The most frequent clinical manifestation of allergic contact dermatitis (ACD) in these patients is the development of painful fissures in the periungual folds. However, lesions may also appear on the face -- particularly the eyelids, cheeks, and lips -- as well as on the neck, likely due to passive transfer via manual contact or airborne exposure [3,26]. Additionally, ACD can be associated with other symptoms, including chronic paronychia, onycholysis, and onychodystrophy in more severe cases. Paresthesia has been reported in 22.5% of affected individuals, while urticaria (1.6%), angioedema, and respiratory distress are less frequently observed [3].

Conversely, four cases of squamous cell carcinoma (SCC) associated with UVA lamp exposure have been documented, without evidence of photodamage in other sun-exposed areas. The latency period between UV exposure and diagnosis ranged from 11 to 15 years [27-29]. Most lamps used for nail curing emit high-intensity UVA radiation (320-400 nm), releasing up to 4.2 times more energy than natural solar radiation within the 355-385 nm wavelength range. The total energy exposure per session ranges from 15 to 22.5 J/m², substantially exceeding the recommended exposure limits established for outdoor workers [30]. In contrast, LED units offer shorter curing times, making them a safer alternative due to the reduced duration of UVA exposure [3].

Limitations

As we have described in this study, high-resolution ultrasound is a valuable diagnostic tool for understanding, diagnosing, and guiding the management of complications arising from the use of acrylic nails and semi-permanent gel polishes. However, this study has several limitations. The small sample size and absence of a control group restrict the generalizability of our findings and preclude definitive conclusions about causality. Additionally, as a descriptive, retrospective study, it primarily identifies associations rather than establishing cause-and-effect relationships. While we detailed the ultrasound technique and the criteria used to identify key complications, we recognize that further studies with larger cohorts, standardized protocols, and more robust statistical analyses are needed to strengthen the evidence base. Despite these limitations, our study provides an important initial framework for exploring these complications and can help guide future prospective investigations in this field.

## Conclusions

This study highlights the association between the use of semi-permanent nail polishes and acrylic nails with various nail complications detected via ultrasound. Users of semi-permanent polishes frequently exhibited thinning of the distal third of the nail plate, loss of the dorsal nail plate, increased thickness of the intermediate plate, and methacrylate deposits within the intermediate plate. Conversely, acrylic nail users demonstrated a higher prevalence of matrix atrophy, ventral pterygium, and nail bed thinning. Nail ultrasound proved to be a valuable diagnostic tool, revealing alterations not always apparent during clinical examination. Furthermore, Doppler imaging identified increased vascularization in all patients, indicating a likely chronic inflammatory response linked to these cosmetic procedures. These findings underscore the importance of thorough diagnostic evaluation and patient education regarding the potential adverse effects of semi-permanent and acrylic nail products. Dermatological monitoring is recommended to facilitate early detection and management of related complications. Despite the limited sample size, this study provides important evidence of nail alterations associated with cosmetic nail use and underscores the need for larger studies to validate these findings and develop preventive strategies.

## References

[1] Pinteala T, Chiriac AE, Rosca I (2017). Nail damage (severe onychodystrophy) induced by acrylate glue: scanning electron microscopy and energy dispersive x-ray investigations. Skin Appendage Disord.

[2] Jefferson J, Rich P (2012). Update on nail cosmetics. Dermatol Ther.

[3] Litaiem N, Baklouti M, Zeglaoui F (2022). Side effects of gel nail polish: a systematic review. Clin Dermatol.

[4] Wortsman X, Alfageme F, Roustan G (2016). Guidelines for performing dermatologic ultrasound examinations by the DERMUS group. J Ultrasound Med.

[5] Gonzalez C, Wortsman X (2024). How to start on dermatologic ultrasound: basic anatomical concepts, guidelines, technical considerations, and best tips. Semin Ultrasound CT MR.

[6] Gonzalez C, Valdivia-Muñoz L (2025). High-frequency ultrasound evaluation of the nail unit: essential insights for clinicians. Cureus.

[7] Vargas EAT, Finato VML, Azulay-Abulafia L (2024). Ultrasound of nails: why, how, when. Semin Ultrasound CT MR.

[8] Sechi A, Wortsman X, Tosti A (2025). Advances in image-based diagnosis of nail disorders. J Eur Acad Dermatol Venereol.

[9] Wortsman X (2021). Concepts, role, and advances on nail imaging. Dermatol Clin.

[10] Wortsman X, Gutierrez M, Saavedra T, Honeyman J (2011). The role of ultrasound in rheumatic skin and nail lesions: a multi-specialist approach. Clin Rheumatol.

[11] Wortsman X (2018). Atlas of Dermatologic Ultrasound. Atlas of dermatologic ultrasound.

[12] Gonzalez C, Barbosa E, Franco MD (2021). High resolution ultrasound of fillers and their implications in aesthetic medicine: a study in human cadavers. Int J Clin Exp Dermatol.

[13] Wortsman X (2015). Identification and complications of cosmetic fillers: sonography first. J Ultrasound Med.

[14] Shihab N, Lim HW (2018). Potential cutaneous carcinogenic risk of exposure to UV nail lamp: a review. Photodermatol Photoimmunol Photomed.

[15] Chessa MA, Iorizzo M, Richert B (2020). Pathogenesis, clinical signs and treatment recommendations in brittle nails: a review. Dermatol Ther (Heidelb).

[16] Chen AF, Chimento SM, Hu S, Sanchez M, Zaiac M, Tosti A (2012). Nail damage from gel polish manicure. J Cosmet Dermatol.

[17] Cervantes J, Sanchez M, Eber AE (2018). Pterygium inversum unguis secondary to gel polish. J Eur Acad Dermatol Venereol.

[18] Hwang S, Kim M, Cho BK (2016). Case of various nail changes induced by gel polish. J Dermatol.

[19] Adigun CG, Shoaf H (2020). Psoriasiform onychodystrophy induced by photobonded acrylic nails. J Clin Aesthet Dermatol.

[20] Mattos Simoes Mendonca M, LaSenna C, Tosti A (2015). Severe onychodystrophy due to allergic contact dermatitis from acrylic nails. Skin Appendage Disord.

[21] Rieder EA, Tosti A (2016). Cosmetically induced disorders of the nail with update on contemporary nail manicures. J Clin Aesthet Dermatol.

[22] Gutierrez M, Wortsman X, Filippucci E (2009). High-frequency sonography in the evaluation of psoriasis: nail and skin involvement. J Ultrasound Med.

[23] Franco MD, González-Díaz CP, Rolón M (2021). Case report of nail psoriasis as the only cutaneous manifestation, a diagnostic challenge. J Colomb Assoc Dermatol Dermatol Surg.

[24] Agache M, Popescu CC, Enache L (2023). Nail ultrasound in psoriasis and psoriatic arthritis-a narrative review. Diagnostics (Basel).

[25] Ramos L, Cabral R, Gonçalo M (2014). Allergic contact dermatitis caused by acrylates and methacrylates--a 7-year study. Contact Dermat.

[26] Muttardi K, White IR, Banerjee P (2016). The burden of allergic contact dermatitis caused by acrylates. Contact Dermat.

[27] Freeman C, Hull C, Sontheimer R (2020). Squamous cell carcinoma of the dorsal hands and feet after repeated exposure to ultraviolet nail lamps. Dermatol Online J.

[28] MacFarlane DF, Alonso CA (2009). Occurrence of nonmelanoma skin cancers on the hands after UV nail light exposure. Arch Dermatol.

[29] Ratycz MC, Lender JA, Gottwald LD (2019). Multiple dorsal hand actinic keratoses and squamous cell carcinomas: a unique presentation following extensive UV nail lamp use. Case Rep Dermatol.

[30] Curtis J, Tanner P, Judd C, Childs B, Hull C, Leachman S (2013). Acrylic nail curing UV lamps: high-intensity exposure warrants further research of skin cancer risk. J Am Acad Dermatol.

